# Interaction Mechanisms and Application of Ozone Micro/Nanobubbles and Nanoparticles: A Review and Perspective

**DOI:** 10.3390/nano12121958

**Published:** 2022-06-07

**Authors:** Wei Xiao, He Zhang, Xiaohuan Wang, Biao Wang, Tao Long, Sha Deng, Wei Yang

**Affiliations:** 1 School of Resources Engineering, Xi’an University of Architecture and Technology, Xi’an 710055, China; hezhangxauat@163.com (H.Z.); longtao@xauat.edu.cn (T.L.); dengsha@xauat.edu.cn (S.D.); yangweixauat@126.com (W.Y.); 2 Western Mining Company Limited, Xining 810002, China; 3 School of Economics and Technology, Anhui Agricultural University, Hefei 230036, China; wxh17733351152@163.com; 4 Anhui Hengyu Environmental Protection Equipment Manufacturing Company Limited, Fuyang 230036, China; wb1056669818@163.com

**Keywords:** ozone micro/nanobubbles, titanium dioxide, phenolic pollutants, photocatalysis, hydroxyl radical

## Abstract

Ozone micro/nanobubbles with catalytic processes are widely used in the treatment of refractory organic wastewater. Micro/nanobubble technology overcomes the limitations of ozone mass transfer and ozone utilization in the application of ozone oxidation, and effectively improves the oxidation efficiency of ozone. The presence of micro/nanobubbles keeps the catalyst particles in a dynamic discrete state, which effectively increases the contact frequency between the catalyst and refractory organic matter and greatly improves the mineralization efficiency of refractory organic matter. This paper expounds on the characteristics and advantages of micro/nanobubble technology and summarizes the synergistic mechanism of microbubble nanoparticles and the mechanism of catalyst ozone micro/nanobubble systems in the treatment of refractory organics. An interaction mechanism of nanoparticles and ozone microbubbles is suggested, and the proposed theories on ozone microbubble systems are discussed with suggestions for future studies on systems of nanoparticles and ozone microbubbles.

## 1. Introduction

In recent years, advanced oxidation methods with green and efficient characteristics have attracted the attention of researchers in the field of water treatment [1,2]. Advanced oxidation methods in the process of water treatment, through the input of light energy, electrical energy and other external energy and $O_{3}$, $H_{2}O_{2}$ and other substances [3], via a series of physical processes and chemical reactions, generate hydroxyl radicals (·OH) and peroxyradical ($O_{2}\cdot^{-}$) active radicals with strong oxidability [4], and the generated hydroxyl radicals can react quickly with electron sites that are rich in organic matter in water, which triggers complex free radical chain reactions, thereby resulting in the degradation and removal of organic matter [5].

As a kind of advanced oxidation technology, ozone catalytic oxidation technology has the characteristics of high oxidation efficiency and no secondary pollution, which can degrade the refractory organic matter in waste and has been widely used in the field of water treatment [6,7,8,9]. However, the low ozone utilization rate and poor mass transfer efficiency in the ozone catalytic oxidation process need to be overcome. Researchers’ strategies for improving ozone catalytic oxidation technology can be divided into two categories: catalyst modification [10,11] and the combination of ozone catalytic oxidation with other water treatment processes [12,13,14]. ${\mathrm{TiO}}_{2}$ nanoparticles (TNPs) have higher catalytic efficiency than plate materials due to their fineness, surface effect and small size effect [15,16].

Micro/nanobubble technology overcomes the limitations of traditional ozone catalytic oxidation technology and effectively improves the utilization rate and mass transfer rate of ozone [17,18]. Micro/nanobubbles can not only accelerate the decomposition of ozone’s hydroxyl radicals, but also release many hydroxyl radicals, which greatly improves the mineralization efficiency of refractory organic compounds [19,20,21]. At the same time, the presence of micro/nanobubbles can also keep the catalyst in a dynamic discrete state, which effectively increases the contact frequency between the catalyst and refractory organic matter and contributes to improving the mineralization efficiency of refractory organic matter [12,22,23].

## 2. Research Status of Phenolic Pollutants

### 2.1. Sources and Hazards of Phenolic Organic Compounds

Phenol and its derivatives are common raw materials and intermediates in China’s industrial production. They are widely used in printing and dyeing [24,25], the chemical industry [26,27] and other applications. Phenolic pollutants are inevitably present in wastewater that is discharged from petrochemical, coking, liquefaction and other industrial production sources [28,29,30,31,32] and have large impacts on human health and the surrounding water environment. According to the national environmental statistics bulletin [33], the discharge of wastewater in China in 2019 was as high as 567.1 tons, of which the discharge of industrial source wastewater that contained volatile phenol was 147.1 tons, which accounted for 25.94% of the total discharge of wastewater. Therefore, the removal of phenolic pollutants from wastewater is the top priority in water environmental treatment.

As the simplest and most basic cyclic hydrocarbon pollutant among phenolic pollutants, phenol is characterized by strong toxicity, good water solubility and difficult natural degradation in environmental water. It is a typical refractory organic pollutant. Phenol and other phenolic pollutants are highly toxic. Phenol in wastewater not only causes damage to the human nervous system, but also leads to headache, anemia and even acute poisoning, which threatens the growth of fish and microorganisms in water [34]. If the content of phenol in the water reaches 5 mg/L, the fish will die of poisoning. At the same time, phenol-containing wastewater also has a severe impact on the aquatic environment. For example, wastewater that contains a high concentration of phenol cannot be used for farmland irrigation; otherwise, it will lead to the reduction and even apoptosis of agricultural products [35]. In view of the toxicity of phenol and other phenolic pollutants, many countries prioritize the prevention and control of water pollution [36]. China’s pollutant discharge standard for urban sewage treatment plants (GB18918-2002) clearly stipulates that the maximum allowable emission concentration (daily average value) of volatile phenol in the discharge of water pollutants from urban sewage treatment plants should not be higher than 0.5 mg/L [37], and the discharge of untreated phenolic wastewater is prohibited.

### 2.2. Treatment of Phenolic Pollutants

With the vigorous development of the coal chemical industry, the petrochemical industry and other industries, the demand for phenolic organics has also increased, and the discharge of phenolic pollutants has brought a large load to the water supply environment. In the process of phenol degradation, various active free radicals [34] are often produced by photocatalysts in the production of intermediate products, such as catechol, hydroquinone and p-benzoquinone, which are finally degraded and mineralized. Efficient degradation and even mineralization of phenol and other phenolic pollutants in wastewater has become a research hotspot in the field of water treatment.

At present, the treatment methods of phenol containing wastewater mainly include physicochemical methods, biochemical methods and advanced oxidation:

(1) Physicochemical methods separate insoluble pollutants in water through mass transfer between different media. They have a good treatment effect on wastewater containing high concentrations of phenol. Because physicochemical methods do not change the chemical properties of the substances in the treatment process, they have the characteristics of simple operation, high system stability and high removal rate [14].
(2) Biochemical methods remove phenolic substances from sewage by domesticating microorganisms with the ability to degrade phenolic pollutants. By domesticating and optimizing the microbial population, using phenolic substances as carbon sources and energy and taking the intake of phenolic substances that are required for their own growth as the degradation mode for degrading phenolic pollutants, biochemical methods provide the advantages of maintaining efficient dominant strains, high treatment efficiency and harmless wastewater treatment [38].
(3) Advanced oxidation methods use oxidation technology with ·OH as the main oxidant. They produce ·OH with strong oxidation using light, electricity or catalysts, which can convert phenolic organics into low-toxicity or nontoxic small molecule organics without selectivity [39]. Advanced oxidation methods have the advantages of a fast reaction rate, complete degradation, no secondary pollution and a wide application range. They are a more effective technology for the treatment of phenolic pollutants [40]. Representative processes include the Fenton oxidation method, electrocatalytic oxidation methods and ozone catalytic oxidation method. Ozone catalytic oxidation methods can be divided into homogeneous catalysis and heterogeneous catalysis according to the type of catalyst. The former decomposes ozone through transition metal ions, and the latter promotes ozone decomposition through solid catalysts [8].

## 3. Ozone Catalytic Oxidation Process

Using the ozone process alone, the utilization rate of ozone is not high, and the mineralization rate of organic matter is low. The combined application of the ozone process and other technologies can achieve low consumption and high efficiency and completely mineralize refractory organic matter. Ozone oxidation with a catalyst is called catalytic ozone oxidation. Catalytic ozone oxidation uses a catalyst to catalyze ozone decomposition to produce ·OH, $\cdot O_{2}{}^{-}$ and other active oxygen free radicals with strong oxygen free radicals to oxidize and degrade organic substances. According to the catalyst type and structure, ozone catalytic oxidation can be divided into homogeneous catalytic oxidation and heterogeneous catalytic oxidation [8].

Many semiconductor materials with photocatalytic properties, such as ${\mathrm{TiO}}_{2}$, ZnO, ZnS, WO_3_ and SnO_2_, are used as photocatalysts [41], but ZnO and CdS are unstable under light, and Zn^2+^ and Cd^2+^ are corrosive, which will result in secondary pollution to the environment. As a photocatalyst, ${\mathrm{TiO}}_{2}$ has become a research hotspot in the field of photocatalytic technology for water treatment because of its nontoxic and harmless nature, strong chemical stability and high catalytic efficiency [42].

### 3.1. Basic Properties of $TiO_{2}$ Nanoparticle Photocatalysts

Due to the refinement of particles, ${\mathrm{TiO}}_{2}$ nanoparticles (TNPs) have surface effects and small-size effects that plate materials do not have [43], which makes the catalytic efficiency of TNP photocatalysts better. ${\mathrm{TiO}}_{2}$ has the following advantages:

(1) High electrocatalytic efficiency. ${\mathrm{TiO}}_{2}$ undergoes an electron transition under light conditions, and the electron hole with strong oxidation that is formed by the electron in the conduction band adsorbs and oxidizes the organic matter and solvent on the semiconductor surface [44].
(2) Excellent chemical stability. ${\mathrm{TiO}}_{2}$ has strong acid and alkali resistance and photochemical corrosion resistance.
(3) Energy saving and low cost. The band gap of ${\mathrm{TiO}}_{2}$ is 3.0–3.2 eV, and the ultraviolet part of natural energy sunlight can be used as the light source.
(4) The reaction conditions are mild, and the final products are ${\mathrm{TiO}}_{2}$, $H_{2}O$ and other harmless substances, which do not produce secondary pollution, and have high potential for energy conservation, conservation and environmental protection.

When ${\mathrm{TiO}}_{2}$ is irradiated by light whose energy is equal to or greater than the band gap width, an electron transition occurs, and the electron ($e^{-}$) on the valence surface excites and transitions to the conduction band [45]. Then, an electron hole pair ($e-h^{+}$) is generated on the valence band, which directly oxidizes and reduces the pollutants that are adsorbed on the surface, or oxidizes the hydroxyl groups (${\mathrm{OH}}^{-}$) on the adsorption surface into hydroxyl radicals (·OH) with strong oxidation and then oxidizes and degrades the organic matter into $H_{2}O$, ${\mathrm{CO}}_{2}$ and other harmless small molecular products [46].

The main mechanism of the photocatalytic reaction is the oxidation of high activity and highly oxidizing ·OH, and the photocatalytic process generates a series of strongly oxidizing free radicals through a free radical chain reaction to realize the mineralization of organic pollutants.

**Figure 1:**
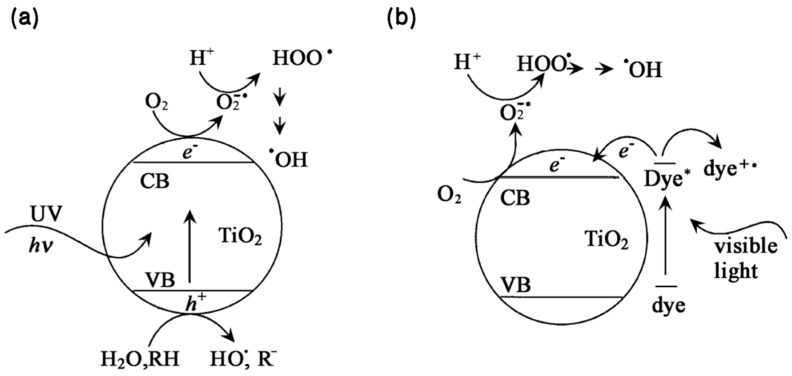
Reaction mechanism diagram of ${\mathrm{TiO}}_{2}$ photocatalytic oxidation under UV (**a**) and visible light (**b**) [47]. Reprinted with permission from Ref. [47].

As illustrated in Figure 1, a series of ${\mathrm{TiO}}_{2}$ reaction processes in photocatalysis can be expressed by the following reaction formulae:

Photoinduced excitation:

$${\mathrm{TiO}}_{2}+h\upsilon \to {\mathrm{TiO}}_{2}+e^{-}+h^{+}$$

Production of ·OH by holes under the action of an electric field:

$${\mathrm{TiO}}_{2}\left(h^{+}\right)+H_{2}O\to {\mathrm{TiO}}_{2}+H^{+}+{\mathrm{OH}}^{-}$$

$${\mathrm{TiO}}_{2}(h^{+})+{\mathrm{OH}}^{-}\to {\mathrm{TiO}}_{2}+\cdot \mathrm{OH}$$

Capture of electrons by oxygen and production of ·OH:

$${\mathrm{TiO}}_{2}(e^{-})+O_{2}\to {\mathrm{TiO}}_{2}+\cdot O_{2}{}^{-}$$

$$H_{2}O+h^{+}\to \cdot \mathrm{OH}+H^{+}$$

$$\cdot O_{2}{}^{-}+H^{+}\to \mathrm{HOO}\cdot$$

$$2\mathrm{HOO}\cdot \to H_{2}O_{2}+O_{2}$$

$$H_{2}O_{2}+e^{-}\to \cdot \mathrm{OH}+{\mathrm{OH}}^{-}$$

$$H_{2}O_{2}+\cdot O_{2}{}^{-}\to \cdot \mathrm{OH}+{\mathrm{OH}}^{-}+O_{2}$$

Oxidization and degradation of the organic matter:

$$\cdot \mathrm{OH}+O_{2}+\mathrm{Organic}\to H_{2}O+{\mathrm{CO}}_{2}+\mathrm{etc}.$$

### 3.2. Application of $TiO_{2}$ Catalyzed Oxidation

The use of ${\mathrm{TiO}}_{2}$ for the photocatalytic degradation of phenolic pollutants has been studied and reported by many scholars. To eliminate the ${\mathrm{TiO}}_{2}$ bottleneck for the further practical application of photocatalysts, researchers have made many efforts to broaden the light response range and improve the quantum conversion efficiency, and a variety of ${\mathrm{TiO}}_{2}$ modification methods [48,49,50] have been put forward to effectively improve the efficiency ${\mathrm{TiO}}_{2}$ in the photocatalytic degradation of phenolic pollutants [51].

(1) Nonmetallic doping modification. Nonmetallic materials are widely available and inexpensive, and nonmetallic ions are doped into the lattice of ${\mathrm{TiO}}_{2}$ to replace the oxygen vacancies of ${\mathrm{TiO}}_{2}$ [52], which can not only reduce the band gap of ${\mathrm{TiO}}_{2}$ nanoparticles and broaden the visible light response range [53,54,55], but also effectively inhibit the recombination of photocarriers [56] and improve their photocatalytic performance.
(2) Surface noble metal deposition modification. When a precious metal is loaded on the surface of ${\mathrm{TiO}}_{2}$, the electrons transfer due to the Fermi energy level [57]: ${\mathrm{TiO}}_{2}$ particles with higher Fermi energy levels will lose electrons and thus gain positive charge, while noble metals will gain negative charge because of electron capture, which makes organic matter more easily photooxidized to secondary holes [58,59,60]. The recombination of holes and photogenerated electrons in the ${\mathrm{TiO}}_{2}$ catalyst can be effectively inhibited [61], thus the transfer efficiency of photogenerated electrons and photocatalytic performance of the ${\mathrm{TiO}}_{2}$ catalyst can be improved.
(3) Oxide composite semiconductor modification. The combination of oxide and ${\mathrm{TiO}}_{2}$ can broaden the light absorption threshold of ${\mathrm{TiO}}_{2}$ [62], effectively improve the separation effect of charge in ${\mathrm{TiO}}_{2}$ semiconductors and improve the photocatalytic activity [63,64] and photocatalytic efficiency [65].

Scholars continue to explore ${\mathrm{TiO}}_{2}$ modifications in order that it can effectively use visible light to reduce the recombination rate of electron holes and to effectively improve the transmission efficiency of photons and enhance ${\mathrm{TiO}}_{2}$ photocatalysis with the objective of improving the organic matter mineralization efficiency of the system.

## 4. Ozone Micro/Nanobubble Technology

In advanced oxidation processes, ozone has strong oxidability and fast reaction speed and causes no secondary pollution to the environment. Therefore, ozone is widely used in drinking water treatment [66,67], printing and dyeing wastewater treatment [6,13,68] and coal chemical wastewater [69,70,71]. Ozone can decompose in water to produce stronger oxidizing substances than itself, such as hydroxyl radicals, which can effectively oxidize and degrade organic pollutants in water [9]. Although ozone has high oxidability, its solubility in water is not high and its stability is poor, which will reduce the elimination and mineralization of refractory organics by ozone [7]. Therefore, improving the solubility and mass transfer efficiency of ozone in water is an important problem to be solved.

### 4.1. Characteristics of Micro/Nanobubbles

Micro/nano bubble refers to the bubble between micron bubble (diameter of 10–50 μm) and nano bubble (diameter of less than 200 nm); different scholars have different definitions of the limit range of its diameter. Generally, bubbles with a diameter of less than 50 μm are considered as micro-nano bubbles, in which microbubbles and nanobubbles are small bubbles with diameters of 10–50 μm and <200 nm, respectively, while bubbles with diameters of more than 50 μm are considered as conventional large bubbles [72,73,74,75,76]. These advantages are mainly reflected in the following aspects:

(1) Large specific surface area. According to the formula S/V = 3/r, the specific surface area per unit volume of a bubble is inversely proportional to the bubble radius. The diameter of a micro/nanobubble is small, and the specific surface area is large. For example, the specific surface area of a bubble with a radius of 1 μm has 1000 times the normal bubble of 1 mm [77]. The larger the specific surface area is, the larger the contact area with the liquid, which corresponds to a higher reaction rate.
(2) Long hysteresis in water. Micro/nanobubbles have smaller diameters than ordinary bubbles. This unique advantage makes them float slowly in the process of gas-liquid mass transfer and have a longer residence time in the liquid [78].
(3) The zeta potential at the gas-liquid interface is high. The surface of a bubble in pure water is rich in negative charges [79]. The zeta potential measured in water of micro/nanobubbles with oxygen as the gas source ranged from −45 to −34 mV, compared to −20 to −17 mV in water of ordinary large bubbles.
(4) Self-rupture produces a mass of free radicals. Micro/bubbles can shrink and burst without external stimulation, and instantly release a large amount of ·OH [80], which has high oxidation potential and can selectively oxidize organic pollutants in water, such as phenol. Because of this characteristic, micro/nanobubbles can be used for the treatment of refractory water.

### 4.2. Ozone Micro/Nanobubble Technology

**Figure 2:**
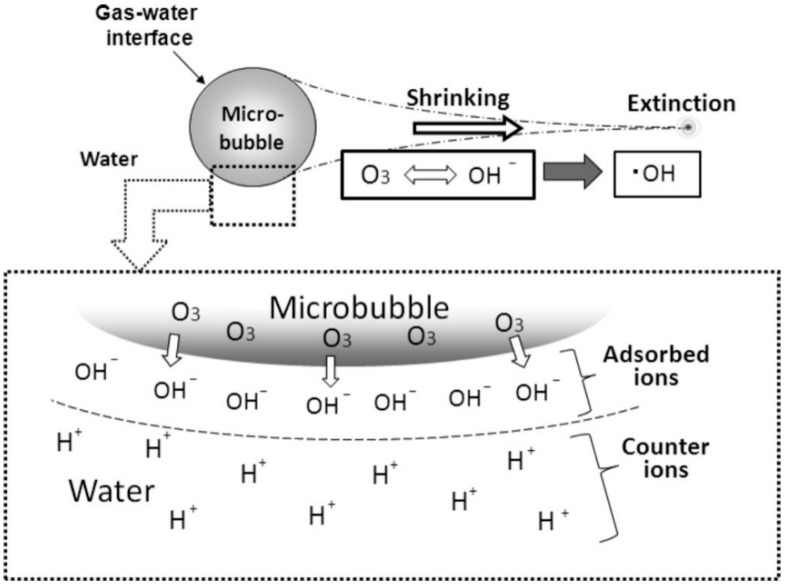
Mechanism of hydroxyl radical production by ozone microbubbles [81]. Reprinted with permission from Ref. [81].

In view of the above characteristics of micro/nanobubbles, a formation diagram of ·OH at the gas-liquid interface of a microbubble [81] is shown in Figure 2. The collapse and autolysis of MNBs and the large accumulation of ions at the gas-liquid interface of bubbles are the key factors for the generation of ·OH. At the same time, the higher compatibility of the bubble surface creates good conditions for the improvement of ozone solubility in $O_{3}$ MNBs; hence, $O_{3}$ MNBs generate more hydroxyl radicals when they break [82].

Hydroxyl radicals have a strong oxidation ability and can quickly react with electronic sites that are rich in organic matter in water, trigger complex free radical chain reactions and degrade most organic matter into $H_{2}O$, ${\mathrm{CO}}_{2}$ and inorganic salts. During the degradation of phenol, ·OH reacts with the electron hole on the phenol molecule to produce the intermediate product hydroquinone and finally mineralize the phenol [83].

### 4.3. Application of Ozone Micro/Nanobubbles to the Degradation of Phenolic Pollutants

The ozone oxidation process has been widely studied by researchers in the field of water treatment [84,85,86], but it is limited by ozone mass transfer and oxidation selectivity in practical applications. Therefore, many researchers have focused on using microbubble processes to enhance the mass transfer efficiency and utilization of ozone [87,88,89].

(1) Microbubbles can effectively improve the mass transfer efficiency of ozone and the yield of ·OH, and then improve the mineralization efficiency of organic matter [18,90,91]. The adjustment of the hydrodynamic behavior of ozone microbubbles can increase the degradation rate of organic matter to realize an obvious removal effect. Micro/nanobubbles can enhance ozone mass transfer and accelerate ozone decomposition to produce ·OH [90]. As the ozone generation rate increases, the partial pressure of ozone also increases, thereby improving the mass transfer of ozone [92].
(2) The oxidation mechanism of ozone microbubbles on organic matter is an indirect oxidation process dominated by the oxidation of free radicals [90,93,94]. Ozone can be oxidized effectively with most organic matter, and micro/nanobubbles can improve oxidation efficiency of ozone to organic matter [90], this is because ozone microbubbles can produce non-selective ·OH, enabling organic matter to achieve more active oxidation degradation [82]. The oxidation of organic matter by ozone microbubbles is an indirect oxidation process dominated by free radical (·OH), which is different from the direct oxidation of organic matter by conventional bubbles [21].
(3) The collapse of micro/nanobubbles can play an important role in the decomposition of organic matter [95,96,97]. Collapsing air micro/nanobubbles can lead to decomposition of phenol, and upon collapse, a large amount of ·OH is released, which plays an important role in the degradation of phenol [98,99]. At the same time, the pH of the solution and the type of gas in the micro/nanobubbles also play an important role in the degradation of phenol. The pH value directly affects the number of free radicals that are generated when an oxygen micro/nanobubble breaks and the degree of ionization of phenol in the aqueous solution [95,98].
(4) Hydroxyl radicals have a higher standard redox potential than ozone and hydrogen peroxide [3,100,101]. The addition of $H_{2}O_{2}$ enhances the formation of ·OH in the system, which may be because the added $H_{2}O_{2}$ oxidant can react with ·OH to form ·OH and promote the formation of ·OH [102]. In addition, it can effectively inhibit the compound reaction of free radicals and enable ·OH to decompose organic matter efficiently [17].

In conclusion, the degradation of phenolic pollutants by the ozone micro/nanobubble method is better than that by traditional bubble methods, which can be combined with other processes to improve the degradation rate of organic compounds.

Micro/nanobubbles have the same characteristics as traditional bubbles. Ozone micro/nanobubble technology can solve the problems of low ozone utilization, selectivity of ozone oxidation and slow gas-liquid mass transfer rate in traditional ozone catalytic oxidation technology for water treatment [82,103]. The presence of microbubbles can accelerate the decomposition of hydroxyl radicals of ozone [17,18] and greatly improve the mineralization efficiency of refractory organics [19,20,21]. Moreover, micro/nanobubbles can also keep the catalyst in a dynamic discrete state, which effectively increases the contact frequency between the catalyst and refractory organics [12,22,23], contributes to the oxidative degradation of refractory organics and gives ozone catalytic oxidation technology broader application prospects in the field of water treatment.

## 5. Catalyst and Micro/Nanobubble Mechanism

### 5.1. Synergistic Interaction between Nanoparticles and Micro/Nanobubbles

When using microbubble processes in combination with photocatalytic oxidation, the catalytic oxidation performance can be promoted without modification or doping of the catalyst, which benefits from the synergy between nanoparticles and microbubbles. Nanobubbles can facilitate nanoparticle substrate adsorption, which occurs by capillary bridge-assisted MNB nanoparticle adhesion, and helps form a contact liner to stabilize bubbles on the three-phase interface [104]. At the same time, MNBs have a high specific surface area, which makes them adsorb with organic matter and interact with nanoparticles on the surface [105].

**Figure 3:**
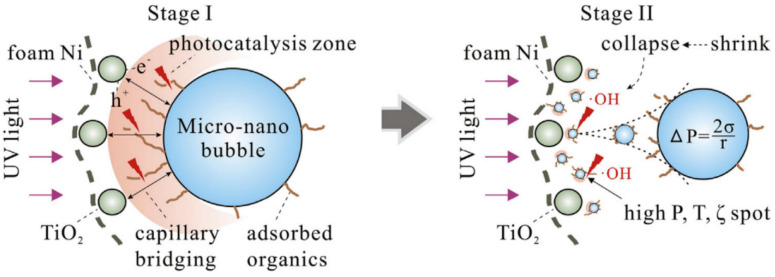
Two stages of purification in the MNB-UV/TiO_2_ hybrid system. P is the pressure, T is the temperature, ζ is the electric potential, ΔP is the Laplace pressure, σ is the surface tension and r is the bubble radius [106] (“foam Ni” is a base material for TiO_2_ attachment). Reprinted with permission from Ref. [106].

Fan et al. [106] evaluated MNB-UV/${\mathrm{TiO}}_{2}$ in the process of advanced treatment of urban secondary wastewater. The purification process in the mixed system consists of two stages (as shown in Figure 3), and these two stages exist synchronously forming a macro perspective. First, MNBs not only have the ability to capture organic pollutants and carriers [107] but also can increase the amount of oxygen in photocatalytic reactions. In the first stage, in the spaces between MNBs and ${\mathrm{TiO}}_{2}$ a photocatalytic region arises that can enhance the degradation of organic matter. The rise, contraction and collapse of bubbles cause MNBs-${\mathrm{TiO}}_{2}$ to form in the second stage of the interaction; the pressure in each bubble increases with the decrease in the bubble’s radius [108]; the MNBs exchange heat very fast with the surroundings; and each bubble has a negative charge over a wide pH range [109]. At this time, the extremely high concentration ion field provides favorable conditions for the generation of strongly oxidizing radicals such as ·OH.

### 5.2. Nanoparticles Promote the Formation and Stabilization of Micro/Nanobubbles

The interaction between the bulk nanobubble solution and nanoparticles occurs through new nucleation on nanoparticles rather than collision [110]. The high-energy barrier and colloidal stability between nanoparticles [111] can effectively prevent their aggregation in dilute solution. At the same time, the addition of nanoparticles can significantly enhance the formation and stability of microbubbles [112] because the gas molecules in the solution are adsorbed on the surface of nanoparticles, and their nucleation is easier than homogeneous nucleation [113].

According to classical nucleation theory [114], the free energy that is required to form a bubble is:

∆G=−V∆p+Aγ

where ∆p is the pressure difference across the interface, γ is the surface tension of the gas–liquid interface, V is the bubble volume and A is the area of the gas-liquid interface. However, for homogeneous nucleation, the free energy that is required to form a spherical bubble with radius *R* is: $∆G=-V∆p+4\pi R^{2}\gamma$. When the bubble reaches equilibrium, the critical radius obeys the Young–Laplace equation: $R^{*}=2\gamma /∆p$. This depends on the bubble size, which is also supported by the findings of Zhang [110] and others that the mixing of nanoparticles and nanobubbles increases the concentration of NBs in the solution and the interaction between plate nanobubbles and nanoparticles.

**Figure 4:**
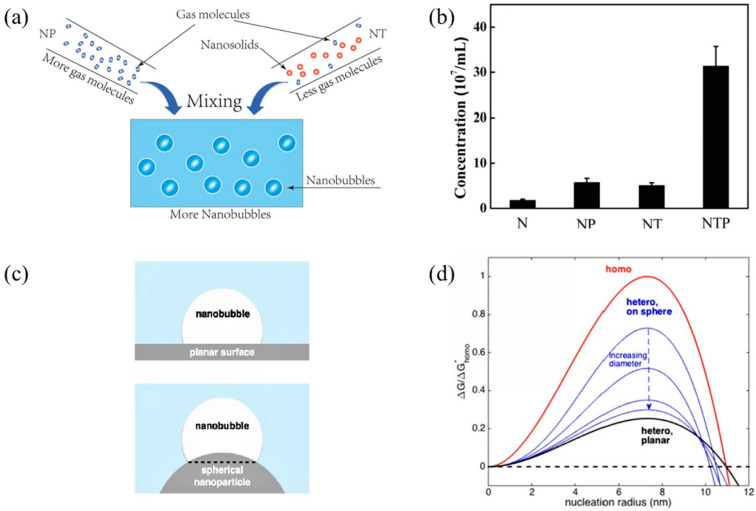
(**a**) A nanoparticles and nanobubbles mixed solution, (**b**) the effect of nanoparticles on nanobubbles, (**c**) the nucleation of nanobubbles at planar and solid/gas interfaces and (**d**) the energy barrier that prevents nucleation for homogeneous nucleation, which shows that nucleation on smaller nanoparticles requires more energy [110,115]. (N—Na_2_CO_3_; NP—pressurized N; NT—Na_2_CO_3_^+^ titanium dioxide; NTP—pressurized NT), reprinted with permission from Refs. [110,115].

As shown in Figure 4, an independent measurement method was established to examine the influence of nanocrystals on NB formation when pure nitrogen was used to produce NBs. Xiao et al. [115] found that, regardless of the pressure, the addition of ${\mathrm{TiO}}_{2}$ nanoparticles can significantly increase the concentration of micro/nanobubbles, and the effect of ${\mathrm{TiO}}_{2}$ nanoparticles on the concentration of micro/nanobubbles is more significant, namely, that the concentration can be increased by 5 times in the pressurized system. A study by Zhang [110] confirmed that the addition of nanosolids can either increase bubble nucleation or provide additional nucleation centers to increase the NB concentration and that pressurization can increase the concentration of dissolved gas in the solution, which can be followed by appropriate decompression to promote homogeneous nucleation of bubbles and, thus, enhance the concentration of bubbles in the solution. The addition of nanosolids can promote the heterogeneous nucleation of bubbles and greatly increase the NB concentration.

## 6. Degradation Mechanism of Organic Matter in the Catalyst-$O_{3}$ Micro/Nanobubble System

### 6.1. Generation of ·OH

**Figure 5:**
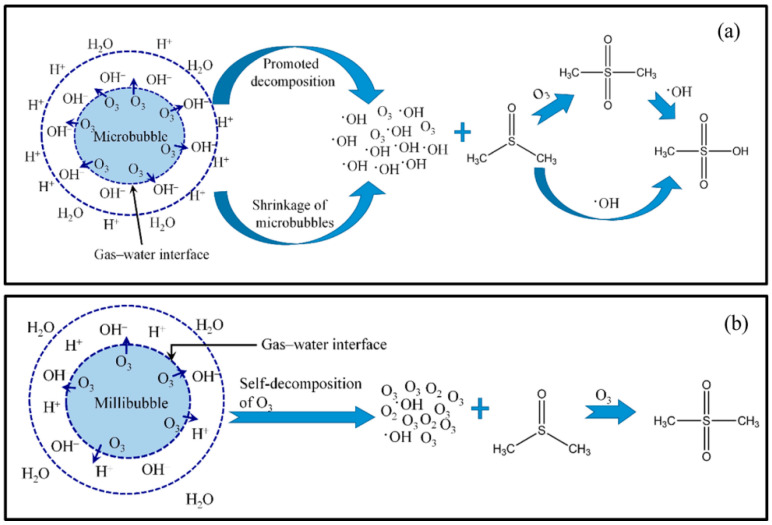
Proposed mechanisms of DMSO degradation by (**a**) OMBs and (**b**) OMLBs [12]. (DMSO—dimethyl sulfoxide; OMBs—ozone microbubbles; OMLBs—ozone millibubbles). Reprinted with permission from Ref. [12].

The presence of microbubbles is an important factor for the conversion of ozone to ·OH. The formation of ·OH in the formed ozone microbubbles is largely due to the increase in the absolute value of the electromotive force at the liquid interface when the microbubbles gradually shrink [116], and large amounts of ${\mathrm{OH}}^{-}$ and $H^{+}$ rapidly accumulate at the bubble interface. Ozone interacts with hydroxyl ions that are adsorbed at the gas-water interface to generate ·OH. The reaction formula is as shown in Figure 5. 

Jabesa et al. [12], based on a comparative test of ozone oxidation under traditional bubbles and oxidative degradation of dimethyl sulfoxide by ozone micro/nanobubbles, proved that the enrichment of ${\mathrm{OH}}^{-}$ on the surface of microbubbles leads to a negative charge on the surface of the microbubble (as shown in Figure 5a), which promotes the self-decomposition of ozone into chiral ·OH. At the same time, the shrinkage and collapse of microbubbles is another important way to produce ·OH. Ozone is transmitted, and the self-decomposition pathway in a bubble system is different from that in a microbubble system (as shown in Figure 5b). Ozone is rapidly decomposed into oxygen due to its instability, forming less ·OH under highly alkaline conditions and less in the traditional bubble system.

### 6.2. Mass Transfer and Decomposition of O_3_

According to Henry’s law, the solubility of ozone is proportional to the partial pressure and total pressure in the system [117]. Although ozone has strong oxidization properties, its available solubility is very small in practical use. Therefore, improving the solubility and mass transfer efficiency of ozone in water is an important problem to be solved. When microbubbles rise in a reactor, they break due to the reduction of their volume and the increase of their specific surface area and surface charge density. The relationship between diameter and pressure can be described by the Young–Laplace equation:

$$P=P_{1}+\frac{4\delta }{d}$$

where P is the gas pressure, $P_{1}$ is the liquid pressure, δ is the liquid apparent tension and d is the bubble diameter.

**Figure 6:**
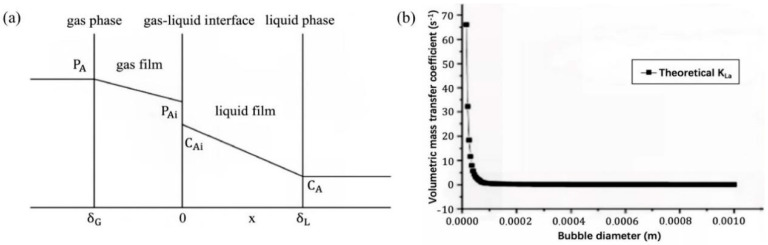
(**a**) A two−mode theoretical diagram of ozone mass transfer [82] and (**b**) the theoretical volumetric mass transfer coefficient values for various bubble diameters [118]. Reprinted with permission from Refs. [82,118].

A larger interfacial area and higher internal pressure endow microbubbles with the potential to improve ozone gas–liquid mass transfer. As shown in Figure 6a, according to the dual-mode theoretical diagram of ozone mass transfer, the gas–liquid interface is located between the gas film and the liquid film, and there is mass transfer resistance at the gas–liquid interface. The relationship between the theoretical mass transfer coefficient and bubble diameter is shown in Figure 6b. When micro/nanobubbles are used for mass transfer, the bubble particle size is in the order of microns and is less than 100 μm. It is generally believed that there is no mass transfer resistance in micro/nanobubbles.

**Figure 7:**
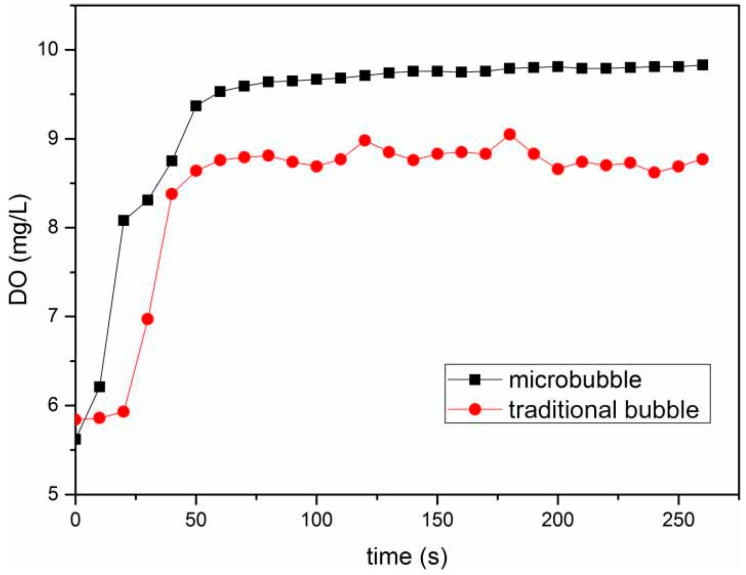
Variation in DO concentration at a gas flow rate of 0.67 L/min [118]. (DO—dissolved oxygen). Reprinted with permission from Ref. [118].

Yao et al. [118] measured and compared the volumetric mass transfer coefficient values of microbubbles and traditional bubbles and demonstrated that microbubbles enhance mass transfer. Under the same gas flow rate, the volume mass transfer coefficient of microbubbles is 32.59% higher than that of conventional bubbles. Figure 7 shows the changes in the dissolved oxygen concentration with time in the microbubble system and in the traditional bubble system. The final stable value of the microbubbles is significantly higher than that of the traditional bubbles.

### 6.3. Synergistic Mechanism

When the catalyst ozone microbubble system oxidizes and degrades organics, there is a synergy between the adsorption of organics by the catalyst and the oxidation of organics by ozone microbubbles, but the degradation of organics in the catalyst ozone microbubble system is not a simple superposition of microbubble ozonation and catalyst adsorption.

**Figure 8:**
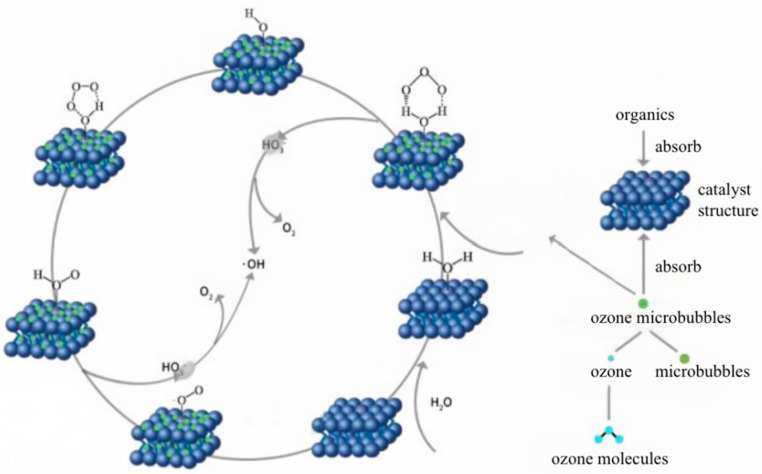
Mechanism of the catalytic process in the MB-catalyst-O_3_ system [120]. Reprinted with permission from Ref. [120].

Zhang et al. [119] proposed four possible synergistic mechanism processes through MB-$O_{3}$-Cu and Mn/${\mathrm{Na}}_{2}$Si$O_{3}$ system experiments (Figure 8): (1) microbubbles adhere to the surface of the catalyst and inside the pores; (2) microbubbles collapse, burst and release large amounts of $O_{3}$ and ·OH; (3) $O_{3}$ reacts at the surface active center of the catalyst to produce ·OH; and (4) oxidative degradation of acid scarlet 3R macromolecular and small organic acids by $O_{3}$ and ·OH occurs. Figure 8 illustrates the catalytic process mechanism of the MB-catalyst-$O_{3}$ system. Oxygen vacancies on the catalyst surface are occupied by adsorbed water molecules to generate –OH_2_^+^ (process 1). Microbubbles adhere to the surface of the catalyst and the insides of the pores, and the collapse and rupture of microbubbles release a large amount of $O_{3}$ and ·OH (process 2). $O_{3}$ and –OH_2_^+^ on the catalyst surface form the six-membered rings under the action of an electrostatic force and hydrogen bonding (process 3). Each six-membered ring decomposes into ·OH due to its instability, and ${\mathrm{HO}}_{3}\cdot$ is further decomposed into OH and $O_{2}$ (process 4). $O_{3}$ and ·OH contact to form a five membered ring (process 5) and then convert to $O_{2}$ and –${\mathrm{HO}}_{2}$– (process 6). $O_{3}$ and –${\mathrm{HO}}_{2}$– react to generate –$O_{2}{}^{-}\cdot$ and ${\mathrm{HO}}_{3}\cdot$ (process 7). $O_{2}$ is released by –$O_{2}{}^{-}$ and the surfactant site is regenerated (process 8).

### 6.4. Additive Effect of $H_{2}O_{2}$

The addition of $H_{2}O_{2}$, which is a strong oxidant, can significantly improve the decomposition rate of organic matter. This is because $H_{2}O_{2}$ photolysis can produce ·OH, which enhances the decomposition of organic matter:

$$H_{2}O_{2}+h\nu \to 2\cdot \mathrm{OH}$$

$H_{2}O_{2}$ can be transformed and quenched with ·OH by the following reaction [121]:

$$2\cdot \mathrm{OH}\to H_{2}O_{2}$$

$$\cdot \mathrm{OH}+H_{2}O_{2}\to {\mathrm{HO}}_{2}\cdot +H_{2}O$$

In the presence of $H_{2}O_{2}$, the ozone oxidation system can generate HO in water and produce ${\mathrm{HO}}_{2}{}^{-}$ and $H_{3}O^{+}$, where ${\mathrm{HO}}_{2}{}^{-}$ reacts again to form ·OH [102] with strong oxidation:

$$O_{3}+H_{2}O_{2}\to \cdot \mathrm{OH}+{\mathrm{HO}}_{2}\cdot +O_{2}$$

$$H_{2}O_{2}+H_{2}O\to {\mathrm{HO}}_{2}{}^{-}+H_{3}O^{+}$$

$$O_{3}+{\mathrm{HO}}_{2}{}^{-}\to O_{2}+O_{2}{}^{-}+\cdot \mathrm{OH}$$

In addition, the surface of rutile ${\mathrm{TiO}}_{2}$ adsorbs $H_{2}O_{2}$ which can be used as a catalyst to promote the formation of ·OH. There is a good correlation between the amount of ·OH that is added to the solution and the apparent oxidation ability; upon exposure to light, ${\mathrm{TiO}}_{2}$ forms surface holes, and ·OH is captured on the ${\mathrm{TiO}}_{2}$ surface. Under neutral pH conditions, ·OH dissociates and adsorbs in the form of trapped holes [10]:

$$\cdot \mathrm{OH}↔\cdot O^{-}+H^{+}$$

$$\cdot \mathrm{OH}+{\mathrm{Ti}}^{4+}↔{\mathrm{Ti}}^{4+}O\cdot^{-}+H^{+}$$

In the rutile ${\mathrm{TiO}}_{2}$/$H_{2}O_{2}$ system, the addition of $H_{2}O_{2}$ greatly increases the content of ·OH, which may be due to ${\mathrm{TiO}}_{2}$-surface-adsorbed $H_{2}O_{2}$, which can promote the generation of ·OH [11,122]. The formation of ·OH by the additive effect of ${\mathrm{TiO}}_{2}$ and $H_{2}O_{2}$ plays a synergistic role in the degradation of organic compounds dominated by ·OH.

In conclusion, the exploration and verification results that are discussed above provide references for the application of catalyst ozone micro/nanobubble systems in the treatment of refractory organics.

## 7. Prospects for Nanoparticles/Ozone Micro/Nanobubbles Systems

Micro/nanobubbles have been widely used in many fields due to their unique properties. Although many excellent results have been obtained and substantial progress has been made in the investigation of catalyst and ozone micro/nanobubbles, this research is still in its infancy, and many challenges remain.

First, the production of controllable MNBs with smaller sizes and higher concentrations is crucial to the application of catalysts and ozone micro/nanobubbles.

Second, the interaction of the catalyst and ozone micro/nanobubbles is limited by the nucleation and collision of micro/nanobubbles. It is necessary to develop new techniques with both higher spatial resolutions and kinetic models.

Third, although several models of the utilization of micro/nanobubbles have been proposed, their long lifetimes remain to be satisfactorily explained. Especially in sludge reduction and river governance, the presence of microbubbles creates an aerobic environment for the sediment, provides conditions for the growth of aerobic bacteria and strengthens the decomposition of organic matter in the sediment to solve the river eutrophication problem.

Finally, it is believed that catalyst/ozone micro/nanobubbles systems have strong prospects in a wide range of applications, especially the degradation of refractory organic matter.

## Data Availability

Not applicable.
